# Phylogenetic and pangenomic analyses of members of the family Micrococcaceae related to a plant-growth-promoting rhizobacterium isolated from the rhizosphere of potato (Solanum tuberosum L.)

**DOI:** 10.18699/vjgb-24-35

**Published:** 2024-06

**Authors:** S.Yu. Shchyogolev, G.L. Burygin, L.A. Dykman, L.Yu. Matora

**Affiliations:** Institute of Biochemistry and Physiology of Plants and Microorganisms – Subdivision of the Saratov Federal Scientific Centre of the Russian Academy of Sciences, Saratov, Russia; Institute of Biochemistry and Physiology of Plants and Microorganisms – Subdivision of the Saratov Federal Scientific Centre of the Russian Academy of Sciences, Saratov, Russia Saratov State Vavilov Agrarian University, Saratov, Russia; Institute of Biochemistry and Physiology of Plants and Microorganisms – Subdivision of the Saratov Federal Scientific Centre of the Russian Academy of Sciences, Saratov, Russia; Institute of Biochemistry and Physiology of Plants and Microorganisms – Subdivision of the Saratov Federal Scientific Centre of the Russian Academy of Sciences, Saratov, Russia

**Keywords:** Arthrobacter, Kocuria, Micrococcaceae, pangenome, PGPR, phylogenetic analysis;, strain verification, Solanum tuberosum L., Arthrobacter, Kocuria, Micrococcaceae, пангеном, PGPR, филогенетический анализ, верификация штаммов, Solanum tuberosum L.

## Abstract

We report the results of taxonomic studies on members of the family Micrococcaceae that, according to the 16S rRNA, internal transcribed spacer 1 (ITS1), average nucleotide identity (ANI), and average amino acid identity (AAI) tests, are related to Kocuria rosea strain RCAM04488, a plant-growth-promoting rhizobacterium (PGPR) isolated from the rhizosphere of potato (Solanum tuberosum L.). In these studies, we used whole-genome phylogenetic tests and pangenomic analysis. According to the ANI > 95 % criterion, several known members of K. salina, K. polaris, and K. rosea (including K. rosea type strain ATCC 186T) that are related most closely to isolate RCAM04488 in the ITS1 test should be assigned to the same species with appropriate strain verification. However, these strains were isolated from strongly contrasting ecological and geographical habitats, which could not but affect their genotypes and phenotypes and which should be taken into account in evaluation of their systematic position. This contradiction was resolved by a pangenomic analysis, which showed that the strains differed strongly in the number of accessory and strain-specific genes determining their individuality and possibly their potential for adaptation to different ecological niches. Similar results were obtained in a full-scale AAI test against the UniProt database (about 250 million records), by using the AAI-profiler program and the proteome of K. rosea strain ATCC 186T as a query. According to the AAI > 65 % criterion, members of the genus Arthrobacter and several other genera belonging to the class Actinomycetes, with a very wide geographical and ecological range of sources of isolation, should be placed into the same genus as Kocuria. Within the paradigm with vertically inherited phylogenetic markers, this could be regarded as a signal for their following taxonomic reclassification. An important factor in this case may be the detailing of the gene composition of the strains and the taxonomic ratios resulting from analysis of the pangenomes of the corresponding clades.

## Abstract (ru)

Исследованы представители семейства Micrococcaceae, родственные, согласно тестам 16S рРНК, ITS1 (транскрибируемый межгенный спейсер), средней нуклеотидной идентичности (ANI) и средней аминокислотной идентичности (AAI), штамму RCAM04488 Kocuria rosea – стимулирующей рост растений ризобактерии (PGPR), изолированному из ризосферы картофеля (Solanum tuberosum L.) с использованием полногеномных филогенетических тестов и пангеномного анализа. Согласно критерию ANI > 95 %, ряд известных представителей видов K. salina, K. polaris и K. rosea (включая типовой штамм K. rosea ATCC 186T), наиболее близкородственных изоляту в тесте ITS1, должны быть приписаны к одному и тому же виду с соответствующей верификацией штаммов. Однако указанные штаммы были выделены из весьма контрастных по экологии и географии мест обитания, что не могло не сказаться на их генотипе и фенотипе и должно быть так или иначе учтено в оценках их систематического положения. Данное противоречие проясняют результаты пангеномного анализа, продемонстрировавшие существенные различия в этих штаммах количества акцессорных и штамм-специфичных генов, определяющих их индивидуальность и, возможно, потенциал для адаптации к различным экологическим нишам с соответствующими фенотипическими признаками. Аналогичные результаты получены в тесте AAI в полномасштабном варианте его применения против базы данных UniProt (около 250 млн записей) с использованием программы AAI-profiler и протеома штамма K. rosea ATCC 186T в качестве запроса. Согласно критерию AAI > 65 %, в один и тот же род с Kocuria должны быть объединены представители рода Arthrobacter и некоторых других родов, относящихся к классу актиномицетов, с весьма широким географическим и экологическим спектром источников их выделения. В рамках парадигмы о вертикально наследуемых филогенетических маркерах это можно трактовать как сигнал для их последующей таксономической переквалификации. Важным фактором при этом может быть детализация генного состава штаммов и таксономических соотношений, получаемых в результате анализа пангеномов соответствующих клад.

## Introduction

The paper (Potanina et al., 2017) presented the results of phylogenetic studies on the plant-growth-promoting (Kargapolova et al., 2017) bacterial strain Kocuria rosea RCAM04488, isolated from surface-sterilized roots of potato (Solanum tuberosum L. ‘Kondor’). For the genotypic taxonomic identification of this isolate, sequences of the 16S rRNA gene (GenBank MF754147.1) and of the ITS1 transcribed intergenic spacer (GenBank MF765458.1) were obtained. By using 16S rRNA (Potanina et al., 2017), the evolutionary proximity of this isolate to the genera Rothia, Arthrobacter, and Zhihengliuella, as well as to members of the species K. rosea and K. polaris, was ascertained.

Kocuria is a genus of gram-positive bacteria of the family Micrococcaceae, phylum Actinobacteria, which are either aerobic or facultatively anaerobic. To date, 32 Kocuria species have been identified.

Kocuria bacteria have been found on human and animal skin and mucous membranes. They are generally considered nonpathogenic but can be detected in some urinary tract infections and in hepatobiliary, cardiovascular, nervous system, and gastrointestinal infections (Kandi et al., 2016). Although Kocuria can infect immunocompromised patients, they are weakly pathogenic and are highly sensitive to antibiotics (Odeberg et al., 2023).

Many Kocuria members, including the type species K. rosea, live in soil (Stackebrandt, Schumann, 2015) and are endophytes; i. e., they have been isolated from the rhizosphere and tissues of many plants. Endophytic Kocuria are inhibitory to several pathogenic fungi and bacteria (Cho et al., 2007; Rao et al., 2015; Andreolli et al., 2016; Candra et al., 2022; Tavarideh et al., 2022; Tedsree et al., 2022). In addition, some of them have properties of plant-growth-promoting rhizobacteria (PGPR), because they produce indole-3-acetic acid and other phytohormones and because they increase plant resistance to stress (Passari et al., 2017; Li et al., 2020).

Bacteriocins from nonlactic acid bacteria, in particular variacin from K. varians, can be used for the biopreservation of food (cheese and meat) products (Gálvez et al., 2010). The K. rosea exopolysaccharide, kocuran, is used in the production of antimicrobial coatings (Kumar, Sujitha, 2014).

A number of soil Kocuria can degrade some xenobiotics, in particular phthalate esters, pesticides, and salts of arsenic, copper, and other heavy metals (Kaur et al., 2015; Román- Ponce et al., 2016; Hansda et al., 2017; Mukherjee et al., 2018; Vital et al., 2019; Yastrebova, Plotnikova, 2020; González- Benítez et al., 2021; Mawang et al., 2021). Various Kocuria have been recovered from soils; marine sediments; meat, dairy and seafood products; beer; seawater; rocks; livestock bedding; manure; surface spring water; and other sources (Church et al., 2020).

The aim of this research was to obtain phylogenetic and genetic information on Micrococcaceae members related to K. rosea isolate RCAM04488 according to the following tests: 16S rRNA (Potanina et al., 2017), internal transcribed spacer (ITS1), average nucleotide identity (ANI), and average amino acid identity (AAI). Whole-genome phylogenetic tests and pangenomic analysis were applied to the known results of whole-genome DNA sequencing of these strains.

## Materials and methods

In our phylogenetic and pangenomic studies, we used the published genomes of the bacterial strains under study, brought into consideration as a result of the use of the bioinformatic resources mentioned below. The characteristics of the genomes are summarized in Results and Discussion and in Supplementary Materials.

The blastn program (Standard Nucleotide BLAST.https://blast.ncbi.nlm.nih.gov/Blast.cgi? PROGRAM=blastn&PAGE_TYPE=BlastSearch&LINK_LOC=blasthome. Accessed 09/06/2023) was used in taxonomic analysis with the genetic sequence of the ITS1 intergenic spacer of K. rosea RCAM04488 (GenBank MF765458.1). Strain RCAM04488 is part of the Russian Collection of Agricultural RCAM04488) and of the Collection of Rhizosphere Microorganisms, Institute of Biochemistry and Physiology of Plants and Microorganisms, Russian Academy of Sciences (IBPPM RAS) http://collection.ibppm.ru,accessed 09/06/2023; IBPPM604).

The average nucleotide identity (ANI) test http://enve-omics.ce.gatech.edu/g-matrix(Goris et al., 2007; Rodriguez-R, Konstantinidis, 2014; Jain et al., 2018), in its OAT modification https://www.ezbiocloud.net/tools/orthoani. (Lee et al., 2016), was used for quantitative species/genus demarcation on the basis of whole-genome sequencing of the strains’ DNA. Note that the 16S rRNA, ITS1, ANI, and AAI tests were developed within the paradigm of vertically inherited prokaryotic genotypic traits by using markers from the core component of the pangenome (Tettelin, Medini, 2020) without any account of the effects of horizontal gene transfer (HGT) in its accessory (optional) and strain-specific parts. The HGT effects largely control the variety of phenotypic traits that determine, in particular, the ability of bacteria and archaea to adapt and function in diverse, frequently changing ecological niches (Koonin, 2012). These traits are taken into account in the analysis of the systematic position of entries that is based on the polyphasic approach, which is very common in the traditional systematics of the prokaryotes (Oren, Garrity, 2014). Hence follows the obvious conventionality of phylogenetic analysis within any scheme using only vertically inherited phylogenetic markers, as do possible contradictions of its results to the traditional classification and nomenclature of the prokaryotes (Shchyogolev, 2021). This probably explains, in particular, the need for their verification with appropriate classification changes, which turned out to be relevant for about 60 % of the Genome Taxonomy Database https://gtdb.ecogenomic.org (GTDB) entries analyzed in Parks et al. (2018)

We used the PGAP program http://pgaweb.vlcc.cn/analyze. (Chen et al., 2018) to obtain information on pangenome composition for selected phylogenetic groups of bacteria (clades).

We used the AAI-profiler program http://ekhidna2.biocenter.helsinki.fi/AAI (Medlar et al., 2018) to evaluate the whole-genome systematic position of Kocuria members relative to the entries from the UniProt protein property database https://www.uniprot.org. (250 million records) for Kocuria members found by the 16S rRNA and ITS1 tests to be closely related to K. rosea RCAM04488. In particular, the program detects and visualizes possible contradictions in the classification of pro- and eukaryotes and microbial contamination (Medlar et al., 2018). To visualize and analyze phylogenetic trees, we used the MEGA11 program https://www.megasoftware.net..

## Results and discussion

Strains closely related to Kocuria rosea isolate RCAM04488 in the ITS1 test

Use of blastn with the sequence of the ITS1 intergenic spacer of K. rosea RCAM04488 (GenBank MF765458.1) against the RefSeq Genome Database (refseq_genomes) with the Kocuria option (taxid:57493) yielded a set of 12 hits. These included ITS1 sequences from Kocuria members and references to the results of whole-genome DNA sequencing of all (mostly type) strains in the set (Supplementary Material 1). Of note, in the BacDive database (Reimer et al., 2022), on the web page https://bacdive.dsmz.de/strain/7641, information is given on 29 entries representing the type strain of K. rosea, including K. rosea DSM 20447T, which, according to the 16S rRNA test, is evolutionarily close to the isolate we are studying (Potanina et al., 2017). Among the results of similar studies conducted by us with the use of the resource https://www.ezbiocloud.net(data not shown), K. rosea strain ATCC 186T (characteristics summarized in Supplementary Material 1) is indicated as the type strain. It is also found on the K. rosea DSM 20447T BacDive web page, presented as the type strain in Trachtenberg et al. (2018), and used for comparison in pangenomic analysis and in AAI-profiler studies


Supplementary Materials are available in the online version of the paper: https://vavilovj-icg.ru/download/pict-2024-28/appx12.pdf


**Fig. 1.**
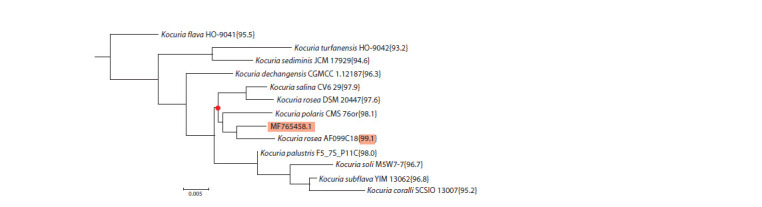
Distance tree for BLAST hits with the ITS1 query sequence (GenBank MF765458.1) of K. rosea RCAM04488 against the RefSeq Genome Database (refseq_genomes) with the Kocuria option (taxid:57493). The identity values (I, %) between sequence MF765458.1 and hit sequences are indicated in curly brackets.

The BLAST distance tree for ITS1 of K. rosea RCAM04488 (GenBank MF765458.1) with the indicated 12 hits (Fig. 1) shows clustering of K. rosea RCAM04488 with members of K. rosea, K. polaris, and K. salina, in agreement with the main results of the 16S rRNA test (Potanina et al., 2017). The value of the ITS1 sequence identity I = 99.1 % among those marked in Figure 1 and the general structure of the cluster, the node of which is marked by a red dot in Figure 1, indicates that K. rosea RCAM04488 is most closely related taxonomically to K. rosea strain AF099C18 in this test. Strain AF099C18 belongs to the type species of the genus Kocuria, the members of which are found in very diverse ecological niches (Stackebrandt, Schumann, 2015) (see Introduction and Supplementary Material 1), and was isolated in Eugene (Oregon, USA) from a dust sample during the study of the effect of the finishing of indoor surfaces on bacterial viability (Hu et al., 2019).

The other members of this cluster include K. rosea strain DSM 20447T, a member of the Actinobacteria; K. polaris type strain CMS 76orT, isolated from cyanobacterial mats in McMurdo Dry Valley, Antarctica (Gundlapally et al., 2015); and K. salina strain CV6 29, isolated in the vicinity of Lake Schott el Djerid (Tunisia) from the roots of Cistanche violacea, a desert plant of the Orobanchaceae family that lives on the roots of host plants (tamarix, black saxaul). The adaptation of these strains to such a contrasting diversity of habitats and conditions can be attributed to the phenotypic traits encoded by the genes in the accessory and strain-specific parts of the Kocuria pangenome, to which a large contribution is probably made by HGT (Treangen, Rocha, 2011; Koonin, 2012).

At the whole-genome level with housekeeping genes, however (ANI test, orthologous genes), all four strains in the monophyletic group with sequence MF765458.1 (Fig. 1) obey the ANI > 95 % condition and, therefore, should be considered as belonging to the same species (Jain et al., 2018). This is illustrated by the ANI dendrogram (UPGMA variant) obtained by the OAT method described in Lee et al. (2016) (Supplementary Material 2). In addition to the four strains listed above, the ANI > 95 % condition, which groups the strains into the same species (Jain et al., 2018), is also obeyed by the K. sediminis JCM 17929T–K. turfanensis HO-9042T pair. Unlike 16S rRNA and ANI, the ITS1 test (Fig. 1) does not have quantitative criteria for grouping/demarcating taxa. In our case, this turned out to be possible only by combining the OrthoANI dendrogram with the heat map in Supplementary Material 2.

To elucidate those whole-genome details that determine the strains’ individuality and possibly also differences in adaptation behavior in contrasting ecological niches, we performed a pangenomic analysis (Chen et al., 2018; Tettelin, Medini, 2020) of the strains included in Figure 1 and Supplementary Material 2 by using the PGAweb program. To the 12 genomes of these strains, we added the genome of K. rosea strain ATCC 186T (see above).

**Fig. 2.**
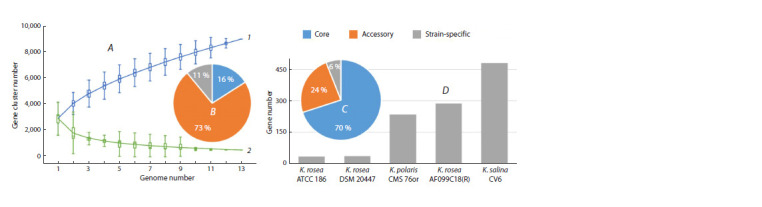
Pangenomic analysis of the Kocuria strains included in Figure 1 and Supplementary Material 2. A, size of the pangenome (1) and core genome (2) versus the number of genomes being considered. B, pie chart of pangenome contents in Fig. A with core, accessory, and strain-specific genes. C, pie chart of the pangenome contents corresponding to the monophyletic group highlighted in Figure 1 (red dot), with core, accessory, and strain-specific genes. D, histogram of the number of strain-specific genes in the strains corresponding to the pangenome in Fig. C.

Figure 2A shows the dependences of pangenome size (curve 1) and core genome size (curve 2) on the number of genomes being considered for the set corresponding to Figure 1. Different colors and numbers in the pie chart of Figure 2B denote the content of the core, accessory, and strain-specific genes in their total pool for a clade of 13 strains. The general appearance of curves 1 and 2 indicates that this pangenome is of the open type, which means that it allows DNA exchange with the global prokaryote gene pool through a variety of mechanisms (Chen et al., 2018; Tettelin, Medini, 2020), including HGT (Treangen, Rocha, 2011; Koonin, 2012).

To elucidate subtle differences among these five genomes, which possibly contribute substantially to their distribution across different ecological niches and other individual phenotypic traits but are not evident in the ANI test (see Supplementary Material 2), we conducted a separate pangenomic analysis for 5 strains corresponding to the monophyletic group highlighted in Figure 1 (red dot) (Fig. 2C, D). For this clade of closely related species, the analysis showed a relatively high content of core genes (70 %), a lower content of accessory genes (24 %), and a low percentage of unique (strain-specific) genes (6 %), with very marked interstrain differences in their number (Fig. 2D).

The largest number of unique genes is present in the endophytic strain K. salina CV6 (480), which is followed by K. rosea AF099C18 (287) and K. polaris CMS 76orT (235). The letter R in K. rosea strain AF099C18 in Figure 2, D shows its status as a representative of K. rosea isolate RCAM04488 at the whole-genome level, which is the most closely related to it in the 16S rRNA (Potanina et al., 2017) and ITS1 tests (Fig. 1).

Strains related to Kocuria rosea RCAM04488 in the AAI-profiler test

Using AAI-profiler, we made an extended whole-genome evaluation of the systematic position of the Kocuria members related to K. rosea RCAM04488 according to the 16S rRNA (Potanina et al., 2017) and ITS1 tests (see above). This was done at the level of the UniProt database, which has about 250 million records as of autumn 2023. With allowance for the close kinship between K. rosea strains, which is shown in Supplementary Material 2, we chose K. rosea type strain ATCC 186T as the initial one for use in AAI-profiler

**Fig. 3.**
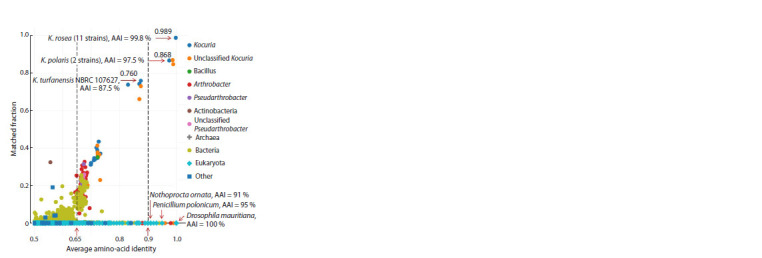
AAI distribution diagram for K. rosea ATCC 186T, as found in the AAI-profiler output data. Explanations are in the text.

The query was the proteome of K. rosea ATCC 186T (genome assembly GCF_006094695.1), used by AAI-profiler to determine AAI between the query proteome and the proteomes of the species members in UniProt and to construct an AAI distribution diagram (Fig. 3). AAI values are plotted on the horizontal axis, and the values of the MF (matched fraction, the proportion of query proteins that have matches in the species analyzed by the program) are plotted on the vertical axis. The diagram icons correspond to the species that received the highest scores, with account taken of AAI and coverage, i. e., the sum of the sequence identity values for all query proteins with established matches

Related species, grouped and colored on the basis of genus, form a characteristic “cloud” in the diagram, with AAI values reflecting the evolutionary closeness of the UniProt strains and the query strain. The horizontal axis has icons for the species for which only individual proteins have been sequenced. The icons are colored according to genus (bacteria) or order (eukaryotes). Eukaryotic species are marked with rhombuses; bacteria, with circles; archaea, with crosses; and everything else (viruses, metagenomes, and unclassified samples), with squares. The vertical dashed lines in Figure 3 correspond to the AAI cutoff values for strain demarcation on the basis of genus (AAI > 0.65) and species (AAI > 0.9) under the conditions presented in the ANI/AAI-Matrix resource, on the websitehttps://help.microbial-genomes.org/ understanding-results#distance , and in Rodriguez-R and Konstantinidis (2014).

The results (Fig. 3) show that the query proteome corresponds to a set of 11 K. rosea strains with average coverage and AAI values of 0.989 and 99.8 %, respectively. The closest to this set among the classified ones is that of two K. polaris strains (including the type strain CMS 76or), the icon of which is located in the area of the diagram with AAI values > 90 % (to the right of the vertical dashed line with an abscissa of 0.9). In the AAI test, this means that all these 13 strains and some other Kocuria members (not shown here) belong to the same species (Luo et al., 2014).

As an example, the Figure 3 diagram includes K. turfanensis strain NBRC 107627T, the icon of which is located within the range of 0.65 <ANI < 0.9, where most species belonging to the same genus in the AAI test (Luo et al., 2014) are concentrated. These include Kocuria of the following species: coralli, flava, indica, marina, palustris, rhizophila, sediminis, soli, subflava, turfanensis, tytonicola, tytonis, and varians. However, the same area in the diagram with coverage in the range 0.12–0.74 also includes members of the genus Arthrobacter (the most widely represented genus in the Fig. 3 diagram) and of the genera Pseudarthrobacter, Micrococcus, Microbacterium, and some other actinomycetes. The placement of these entries (and other bacterial species/strains marked with pink, green, and light green dots) into the main cluster of the genus Kocuria, which groups species related to the query strain K. rosea ATCC 186T (to the right of the dashed line with abscissa AAI = 0.65), could be interpreted as a signal for their probable taxonomic reclassification (Medlar et al., 2018) within the paradigm with vertically inherited phylogenetic markers (Koonin, 2012). However, a decision on this should be made after additional genotypic and phenotypic features are considered within a polyphasic approach (Oren, Garrity, 2014). These strains are listed together with their detailed characteristics in the AAI-profiler output

Six icons corresponding to eukaryotes are located on the abscissa axis at the intraspecies level with AAI values > 0.9. The relatively small (symbolically zero) coverage values mean that only individual proteins have been sequenced for them (Medlar et al., 2018). The protein system from the fruit fly Drosophila mauritiana proved closest to that of K. rosea ATCC 186T on the basis of AAI = 100 %. Next in descending order of AAI values in the intraspecies interval 90 % < AAI < 100 % (Luo et al., 2014) are Penicillium polonicum (imperfect fungus), Poeciliopsis prolifica (small freshwater fish), Drosophila sechellia (another fruit fly species), Nothoprocta ornata (flightless bird), and Hirsutella minnesotensis (asexual propagating fungus). For clarity, some of these are marked with arrows in Figure 3. In the same ANI > 90 % interval, the icons for members of the prokaryotes and the genera Kocuria, Arthrobacter, Micrococcus (all actinomycetes), and Nitrosomonas (β-proteobacteria) are shown on the abscissa axis.

For prokaryotes, this is explained by HGT (Treangen, Rocha, 2011; Medlar et al., 2018). For eukaryotes, which in our case include members of the animal and fungal kingdoms, the high homology between their protein systems and those of the genus Kocuria (class Actinomycetia) may be associated with symbiogenesis as a very probable mechanism of the origin of eukaryotes with the participation of prokaryotes (Dey et al., 2016; Provorov et al., 2018). Bioinformatic studies of this phenomenon have been reported, for example, in Markov and Kulikov (2005) and in Nikitin (2016). They provide data to show that although archaea [from which a considerable part of the eukaryotic genome originates (Stairs, Ettema, 2020)], α-proteobacteria (precursors to mitochondria), and cyanobacteria (precursors to plastids) play fundamental parts in symbiogenesis, substantial contributions to these processes are made by various bacteria, not limited to the above two taxa.

**Fig. 4.**
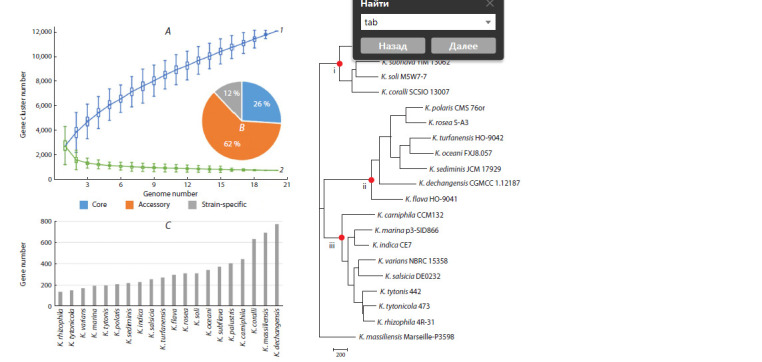
Results of pangenomic analysis of Kocuria strains with the reference genomes, listed in Supplementary Material 3. A, size of the pangenome (1) and core genome (2) versus the number of genomes being considered. B, pie chart of pangenome contents in Fig. A with core, accessory, and strainspecific genes. C, histogram of the number of strain-specific genes in the strains corresponding to the pangenome in Figs. A and B. D, phylogram for the set of strains from the pangenome in Figs. A–C. The phylogram was obtained by the NJ method on the basis of the gene acquisition/loss matrix. Red dots and Roman numerals indicate nodes of monophyletic groups.

In the context of the above-mentioned AAI-profiler results, of interest is the information on the general genomic structure of Kocuria and Arthrobacter members. This information was obtained by pangenomic analysis with the PGAweb software package described in Chen et al. (2018). In the database of the results of whole-genome DNA sequencing of prokaryotic strains https://www.ncbi.nlm.nih.gov/datasets/genome, as of autumn 2023, we found a fairly representative set of genomes for 20 mostly type strains of Kocuria species, having the status of reference genomes (Supplementary Material 3) and used by us for pangenomic analysis (Fig. 4).

The Figure 4 results show the overall conservatism of the genomes being considered (26 % of the core genes) and the pronounced openness of the pangenome (Fig. 4A, B, curves 1 and 2). The changes in the accessory (62 % of the total number of genes) and strain-specific (Fig. 4C) components of the pangenome, which reflect the species diversity of Kocuria, can also be attributed to the great diversity of habitats of these strains – from animal and plant organs and tissues to food, soil, air, and marine environments, including Antarctic cyanobacterial mats

Figure 4D shows the phylogram presented in the final PGAweb results. It was obtained by the neighbor-joining (NJ) method on the basis of the gene acquisition/loss matrix for the pangenome as a whole (Chen et al., 2018). This tree shows a clear distribution of strains over three monophyletic groups (Fig. 4D, red dots). There is no sufficiently pronounced dependence on geographical or environmental factors. It can only be noted that strains of animal origin are concentrated in Figure 4D in group iii.

For comparison and with account taken of the wide representation of Arthrobacter species in the AAI-profiler output (Fig. 3), we also performed a pangenomic analysis for the species of this genus corresponding to these members. The obtained set of predominantly type strains and genomes in the “Reference genomes” category, which we found in the Genome database, is presented in Supplementary Material 4.

**Fig. 5.**
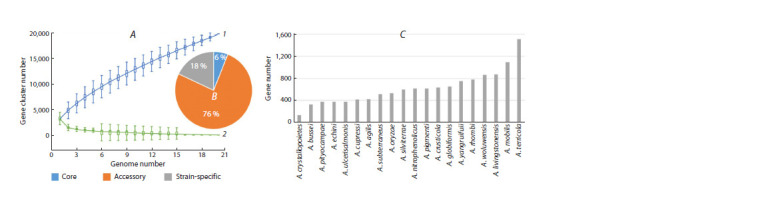
Results of pangenomic analysis of Arthrobacter strains with reference genomes, listed in Supplementary Material 4. A, size of the pangenome (1) and core genome (2) versus the number of genomes being considered. B, pie chart of pangenome contents in Fig. A with core, accessory, and strainspecific genomes. C, histogram of the number of strain-specific genes in the strains corresponding to the pangenome in Figs. A and B.

Figure 5A, B shows the pangenome characteristics for the group of Arthrobacter species listed in Supplementary Material 4. Note that the relative number of core genes for the Arthrobacter group is much smaller than that for the Kocuria group (Supplementary Material 3, Fig. 4). The greater number of accessory and strain-specific genes in Arthrobacter (94 %), as compared with Kocuria (74 %), indicates higher overall genomic heterogeneity of the Arthrobacter group under consideration and greater openness of its pangenome. The number of strain-specific genes in each of the strains forming part of the Arthrobacter pangenome group varies in a wide range, from 130 (A. crystallopoietes) to 1,517 (A. terricola) (Fig. 5C).

## Conclusion

We have shown that, according to the ANI test, the strains K. salina CV6 and K. polaris CMS 76orT, together with K. rosea DSM 20447, K. rosea AF099C18, and K. rosea ATCC 186T, formally within a phylogeny with vertically inherited markers, should be assigned to the same species (ANI > 95 %) with appropriate species verification of the strains. Because all five strains have been isolated from strongly contrasting ecological and geographical habitats, this fact could not but affect their genotypes and phenotypes and should be taken into account in the analysis of their systematic position.

We have clarified this contradiction by pangenomic analysis of a clade of 13 Kocuria strains closely related in the 16S rRNA and ITS1 tests to the K. rosea strain of interest, RCAM04488, isolated from surface-sterilized potato roots. The clade includes the above-mentioned Kocuria strains. The analysis has shown the pangenome to be of the open type and has revealed large differences between the above strains in the content of accessory and strain-specific genes, which determine their individuality and possibly potential for adaptation to different ecological niches with the corresponding phenotypic traits. The largest number of unique genes, which are listed in the output of the PGAP program, was observed in the endophytic strain K. salina CV6 (480). This strain is followed by K. rosea AF099C18 (287), which is most closely related to K. rosea RCAM04488 in the 16S rRNA and ITS1 tests. These observations seem important for evaluating the possible gene content of K. rosea RCAM04488 in terms of its abilities as a PGPR. This will be the subject of our further work, which will use the results of whole-genome DNA sequencing of this strain.

Using AAI-profiler, we obtained similar results in a fullscale AAI test against the UniProt database (approximately 250 million records). In particular, these results confirm the need to assign K. rosea and K. polaris members and several other members of the genus Kocuria to the same species (AAI > 90 %). In the phylogenetic aspect, our most substantial finding is the established association of Kocuria, Arthrobacter (the genus most widely represented in these results), Pseudarthrobacter, Micrococcus, Microbacterium, and several other genera as members of the same genus according to the AAI > 65 % criterion. Within a paradigm with vertically inherited phylogenetic markers, this could be regarded as a signal for the following taxonomic reclassification of these entries. In this respect, it may help to comparatively evaluate their gene content and taxonomic relationships on the basis of pangenomic studies. However, to make this responsible decision, one should consider additional genotypic and phenotypic characteristics of the strains under study within a polyphasic approach

## Conflict of interest

The authors declare no conflict of interest.
